# Simultaneous determination of phagocytosis of *Plasmodium falciparum*-parasitized and non-parasitized red blood cells by flow cytometry

**DOI:** 10.1186/1475-2875-11-428

**Published:** 2012-12-21

**Authors:** Valentina Gallo, Oleksii A Skorokhod, Evelin Schwarzer, Paolo Arese

**Affiliations:** 1 Department of Genetics, Biology and Biochemistry, University of Torino, Torino, Italy

**Keywords:** Malaria anaemia, Phagocytosis method, THP-1 cells, Phagocyte

## Abstract

**Background:**

Severe *falciparum* malaria anaemia (SMA) is a frequent cause of mortality in children and pregnant women. The most important determinant of SMA appears to be the loss of non-parasitized red blood cells (np-RBCs) in excess of loss of parasitized (p-) RBCs at schizogony. Based on data from acute SMA where excretion of haemoglobin in urine and increased plasma haemoglobin represented respectively less than 1% and 0.5% of total Hb loss, phagocytosis appears to be the predominant mechanism of removal of np- and p-RBC.

Estimates indicate that np-RBCs are cleared in approximately 10-fold excess compared to p-RBCs. An even larger removal of np-RBCs has been described in *vivax* malaria anaemia. Estimates were based on two single studies both performed on neurosyphilitic patients who underwent malaria therapy. As the share of np-RBC removal is likely to vary between wide limits, it is important to assess the contribution of both np- and p-RBC populations to overall RBC loss, and disclose the mechanism of such variability. As available methods do not discriminate between the removal of np- *vs* p-RBCs, the purpose of this study was to set up a system allowing the simultaneous determination of phagocytosis of p- and np-RBC in the same sample.

**Methods and Results:**

Phagocytosis of p- and np-RBCs was quantified in the same sample using double-labelled target cells and the human phagocytic cell-line THP-1, pre-activated by TNF and IFN*γ* to enhance their phagocytic activity. Target RBCs were double-labelled with fluorescent carboxyfluorescein-succinimidyl ester (CF-SE) and the DNA label ethidium bromide (EB). EB, a DNA label, allowed to discriminate p-RBCs that contain parasitic DNA from the np-RBCs devoid of DNA. FACS analysis of THP-1 cells fed with double-labelled RBCs showed that p- and np-RBCs were phagocytosed in different proportions in relation to parasitaemia.

**Conclusions:**

The assay allowed the analysis of phagocytosis rapidly and with low subjective error, and the differentiation between phagocytosed p- and np-RBCs in the same sample. The presented method may help to analyse the factors or conditions that modulate the share of np-RBC removal *in vitro* and *in vivo* and lead to a better understanding of the pathogenesis of SMA.

## Background

Severe malaria anaemia (SMA) is a frequent cause of mortality mainly in children and pregnant women [1-3]. The most important determinant of SMA appears to be the removal of non-parasitized red blood cells (np-RBCs) [4,5] most likely by intra- and extravascular phagocytosis [1].

The phagocytosis assay presented here utilizes the activated human phagocytic cell line THP-1 and the cytofluorimetric analysis of ingested p-RBCs and np-RBCs double-labelled with the fluorescent carboxyfluorescein-succinimidyl ester (CF-SE) and the DNA label ethidium bromide (EB) to determine phagocytosed np- and p-RBCs, respectively, in the same sample. The assay is very sensitive, can be performed rapidly with low numbers of p- and np-RBCs and overcomes the limitation of currently available methods that do not differentiate between phagocytosis of p-RBCs *vs* np-RBCs.

In spite of the lack of quantitative data on the relative role of phagocytosis of p- *vs* np-RBCs, the presented method may help to clarify the pathogenesis of SMA analysing the factors or conditions that induce and modulate the removal of np-RBC *in vitro* and *in vivo* in both *falciparum* and *vivax* malaria.

## Methods

### Materials

Unless otherwise indicated all reagents were from Sigma-Aldrich, St Louis, MO, USA. Penicillin-streptomycin was from Invitrogen, Carlsbad, CA, USA; TNF was from PeproTech Inc, Rocky Hill, NJ, USA; IFN*γ* was from R&D Systems, Minneapolis, MN, USA; bis[sulphosuccinimidyl]suberate, sodium salt (BS^3^) was from Pierce Biotechnology Inc, Rockford, IL, USA; carboxyfluorescein diacetate succinimidyl ester (CFDA-SE), the non-fluorescent precursor of CF-SE, was from Fluka, Sigma-Aldrich, Milano, Italy; anti-D IgG (Rhophylac®) were from ZLB Behring SpA, Milano, Italy; Ficoll was from Biochrom AG, Berlin, Germany.

### Cultivation of *Plasmodium falciparum* and stage-dependent separation of parasites

*Plasmodium falciparum* parasites (Palo Alto strain, *Mycoplasma*-free) were cultivated in RBCs from healthy donors at 2% haematocrit and synchronized as described [6]. Briefly, schizont-stage parasitized RBCs (para-sitaemia >95%) were mixed for invasion with washed RBCs and kept in growth medium (RPMI 1640 containing 25 mM HEPES, 30 mM glucose, 2 mM glutamine, 0.025 mM adenine, 24 mM NaHCO_3_, 32 mg/l gentamicin and 10% (vol/vol) A^+^ heat-inactivated human plasma) (time 0). After 20 hr incubation in a humidified CO_2_/air incubator, the ring-enriched fraction was separated on and collected from a discontinuous 40/80/90% vol/vol Percoll gradient containing mannitol (6% wt/vol). After further 20 hr incubation under the same conditions, the trophozoite (troph)-enriched fraction was separated and collected as described before. Alternatively, ring- and troph-stages were separated by the same procedure from non-synchronized cultures of p-RBCs and enriched to 60% and 90% parasitaemia, respectively. When needed, the culture was passed through 40% (vol/vol) Percoll containing 6% mannitol (wt/vol) to remove residual bodies (RBs). Parasitaemia was assessed by light microscopy after Diff-Quik® Fix staining (Medion Diagnostics GmbH, Düdingen, Switzerland). Control np-RBCs were incubated and treated in a similar way without schizont inoculation at time 0.

### Fluorescent labelling of p- and np-RBCs with CF-SE and ethidium bromide (EB)

15 μl of packed p- or np-RBCs were suspended in 30 ml of phosphate buffered saline-glucose (PBS-G; 150 mM NaCl, 10 mM Na_2_HPO_4_/NaH_2_PO_4_, 2 mM glucose, pH 7.4) containing 0.2 mM carboxyfluorescein diacetate succinimidyl ester (CFDA-SE), the non fluorescent precursor of CF-SE, and incubated for 10 min at 37°C. CFDA-SE is a non-fluorescent compound that passively diffuses into RBCs where it is de-acetylated to the high fluorescent CF-SE and stably retained within the RBC upon covalent binding to intracellular amino groups [7]. The labelling reaction was stopped by adding 15 ml of heat-inactivated foetal bovine serum (FBS) for 5 min. Thereafter the cells were washed three times with PBS-G and finally re-suspended in PBS-G for opsonization. EB labelling was performed by incubating 15 μl of packed p- or np-RBCs in 135 μl of PBS-G containing 1 mg/ml EB for 20 min at room temperature. The cells were then washed five times with PBS-G. In double labelling experiments p- or np-RBCs were incubated first with EB 1 mg/ml and then with CFDA-SE 0.2 mM as described above. In order to minimize emission overlap of the two fluorochromes, a minor electronic compensation was applied.

### p- and np-RBC opsonization

For phagocytosis, labelled p-and np-RBC, were opsonized with fresh homologous serum from healthy donors. RBCs were suspended at 33% haematocrit in fresh serum, diluted 1:1 (vol/vol) with PBS-G, incubated for 1 hr at 37°C and subsequently washed twice and resuspended in PBS-G for the phagocytosis assay.

### Phagocytosis of positive control RBCs

(a) Opsonization of np-RBCs with anti-D IgG: freshly drawn heparinized human blood was sedimented by centrifugation at 1,180 ***g*** for 5 min at room temperature and mononuclear cells, platelets and neutrophils removed by aspiration. Isolated RBCs were washed twice with PBS-G, labelled with CF-SE as indicated before, resuspended at 33% haematocrit with anti-D IgG diluted 1:64 (vol/vol) with PBS-G, and incubated for 30 min at 37°C. Negative control RBCs were incubated in PBS-G only. After opsonization, RBCs were washed twice and re-suspended in PBS-G for the phagocytosis assay.

(b) Treatment of np-RBCs with zinc/BS^3^: washed RBCs (see preceding section) were re-suspended at 10% haematocrit in HEPES-saline buffer (10 mM HEPES, 140 mM NaCl, 5 mM glucose, pH 7.4) containing 1 mM ZnCl_2_ and incubated for 10 min at room temperature. Thereafter the cross-linking agent BS^3^ (200 mM stock solution in dimethyl sulfoxide) was added to the RBC suspension to a final concentration of 1 mM. After 10 min incubation at room temperature, RBCs were washed twice in HEPES-saline buffer supplemented with 10 mM ethanolamine and then twice in HEPES-saline buffer supplemented with bovine serum-albumin (BSA, 1% wt/vol). Washed zinc/BS^3^-treated RBCs and untreated control RBCs were then labelled with CF-SE, opsonized and resuspended in PBS-G for the phagocytosis assay.

### THP-1 cell cultivation and phagocytosis assay by flow cytometry

Human monocytic THP-1 cells were grown in RPMI 1640 medium supplemented with 10% (vol/vol) heat-inactivated foetal bovine serum (FBS), 2 mM L-glutamine, 100 U/ml penicillin and 100 μg/ml streptomycin. Fresh medium was replaced twice a week. For general maintenance, the cells were seeded at 1 x 10^5^ cells/ml. For phagocytosis, THP-1 cells were pre-activated by TNF (250 U/ml) and IFN*γ* (50 U/ml) for 24 hr in complete RPMI-1640 medium (RPMI 1640 medium supplemented with 10% (vol/vol) heat-inactivated FBS, 2 mM L-glutamine, 100 U/ml penicillin and 100 μg/ml streptomycin) in a tissue culture polystyrene flask (Falcon, BD Biosciences, San Jose, CA, USA). Thereafter cells were harvested and washed three times with RPMI-1640 medium and finally re-suspended in phagocytosis medium (RPMI 1640 medium supplemented with 10% (vol/vol) FBS), to a concentration of 3 × 10^5^ cells/300 μl medium. Pre-activated THP-1 cells were incubated for 150 min with RBCs (1.5 μl packed RBCs per 3 × 10^5^ THP-1 cells) in 300 μl of phagocytosis medium, into round-bottomed 5 ml polypropylene tubes (Falcon BD Biosciences) in a humidified 5% CO_2_ incubator at 37°C. As a negative control, THP-1 cells were incubated in phagocytosis medium, with untreated RBCs kept at the same cultivation conditions. At the end of the phago-cytosis period, the cell suspension was stratified on Ficoll (1 ml) and subsequently centrifuged for 20 min at 914 ***g*** to separate THP-1 cells from not-phagocytosed RBCs. The THP-1 cells were harvested, washed once with RPMI 1640 medium, and resuspended in phagocytosis medium for analysis of phagocytosis by flow cytometry. The fluorescence of THP1 was acquired on a FACSCalibur flow cytometer (BD Biosciences, Sunnyvale, CA, USA) using the Cell Quest software and the files obtained were analysed with the WinMDI (The Scripps Research Institute, La Jolla, CA, USA) software. After gating the THP-1 cell population (physical parameters analysis) excluding contaminating not-phagocytosed RBCs and cell debris, phagocytosis was quantified by fluorescence intensity of the selected cell population. A total of at least 30,000 events were collected per gate for each sample. First, the percentage of phagocytically active THP-1 cells (phTHP-1) was assessed by counting the cells positive for CF-SE fluorescence. Secondly, the percentage of THP-1 cells that have phagocytosed p-RBC (EB positive cells) was quantified. Thirdly, the number of RBCs phagocytosed per phTHP-1 was quantified by comparing the mean fluorescence intensity (MFI) value of a phTHP-1 cell to the MFI value of a labelled RBC.

### Statistical analysis

Independent t-tests were performed to compare percentages of phTHP-1 or numbers of RBCs phagocytosed per phTHP-1 in different np- and p-RBCs populations. P-values (<0.02, <0.002 and <0.001) were used to define statistical significance and shown when appropriate.

## Results

### Optimization of phagocytosis assay

#### THP-1 pre-activation by TNF and IFNγ treatment

The human monocytic THP-1 cells were selected as phagocytes because they express Fc and C3b receptors [8], both essential for phagocytosis of variously damaged or modified np-RBCs as well as p-RBCs [9]. In addition, THP-1 cells display constant characteristics over time, are easily cultivated and not expensive in their maintenance. The intrinsically low phagocytic activity of THP-1 cells was enhanced by pre-treatment with TNF and IFN*γ*, which increase phagocytic activity by upregulating Fc- and complement receptor expression without modifying phagocytic selectivity [10,11]. Pre-treatment with cytokines was optimized at 250 U TNF and 50 U IFN*γ* per ml THP-1 cell suspension (1 x 10^5^ cells/ml) supplemented 24 hr before starting the phagocytosis experiments. To minimize the phagocytosis-unrelated entrance into the phagocytes of non-activated CFDA-SE leaking out of the labelled RBCs [7] the phagocytosis time was shortened to 150 min obtaining a high phagocytosis rate as well as a negligible low non-phagocytosis-specific staining of the phagocytes. The RBC/THP-1 ratio was optimized at 50/1, respectively ( Additional file 1A-B), resulting in at least 90% of maximal phagocytosis and low fluorescence background.

#### Fluorescent labelling of np- and p-RBCs with CF-SE

**Figure 1:**
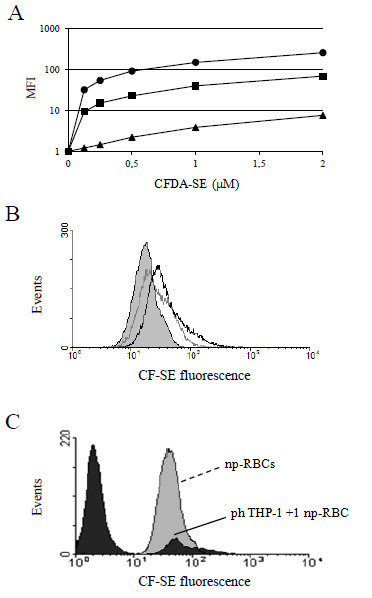
**Fluorescent labelling of np- and p-RBCs with CF-SE. (A)** Dependence of MFI on CFDA-SE concentration (μM final) and haematocrit in np-RBC. (*black circle*) 0.05%, (*black square*) 0.5%, (*black triangle*) 5% haematocrit, corresponding to 5 x 10^3^, 5 x 10^4^, 5 x 10^5^ RBCs/μl, respectively. **(B)** CF-SE fluorescence of np-RBCs (full grey histogram), ring-parasitized RBCs (light grey line), and troph-parasitized RBCs (dark grey line). np- and p-RBCs were incubated as indicated in **(A)**. **(C)** Absence of fluorescence quenching in np-RBCs after phagocytosis. Fluorescence of CF-SE-labelled uningested np-RBCs (full grey histogram, MFI: 48.3 ± 1.6, N = 3) and of phTHP-1 cells fed with limiting numbers of CF-SE-labelled np-RBCs (RBCs/THP-1 ratio = 1, full black histogram, MFI: 49.7 ± 1.1, N = 3) were very similar. For details, see Methods.

Np- and p-RBCs were incubated with CFDA-SE, a non-fluorescent compound that passively diffuses into RBCs where it is de-acetylated to the highly fluorescent CF-SE (carboxyfluorescein succinimidyl ester) and stably retained within the RBC upon covalent binding to intracellular amino groups [7]. Fluorescent RBC labelling reached a plateau after about 10 min and was dependent on CFDA-SE concentration and haematocrit of the RBCs suspension (Figure 1A). Labelling conditions were experimentally optimized at 0.2 mM CFDA-SE, 5,000 RBC/μl corresponding to 0.05% haematocrit and 10 min incubation time. Excess of extracellular protein added after incubation stops further label entrance into RBCs. (see Methods for details). A typical labelling experiment of np-RBC, ring- and troph-parasitized RBCs is shown in Figure 1B. Fluorescence intensity was substantially similar in np- and ring-parasitized RBCs, and distinctly higher in troph-parasitized RBCs, possibly due to an increased uptake of the CFDA-SE or higher esterase activity in late stages of parasite development.

#### Absence of fluorescence quenching after phagocytosis

Quenching of the fluorescence output of CF-SE labelled RBCs after phagocytosis by THP-1 cells was checked. To this end THP-1 cells were fed with limiting numbers of anti-D IgG-opsonized np-RBCs (np-RBCs/THP-1 ratio 1:1, Figure 1C), assuming that on average each THP-1 cell had phagocytosed one np-RBC, as evidenced by the intensity peaks shown in Figure 1C. The MFI of the THP-1 population containing one RBC was compared with the MFI of the labelled uningested np-RBCs. The fluorescence intensity of the two populations was similar: 49.7 ± 1.1 (N = 3) and 48.3 ± 1.6 (N = 3) for THP-1 that have ingested one RBC and uningested RBCs, respectively. Similar results were obtained with p-RBCs (not shown), allowing the conclusion that the fluorescence intensity was not modified within the phagocyte.

#### Phagocytosis of positive control np-RBCs

The method was first checked with positive control np-RBCs. Two RBC treatments were utilized: first, np-RBCs were opsonized with anti-D IgG to stimulate IgG-mediated recognition and phagocytosis via Fc-receptors by the phagocyte. Second, np-RBCs were treated with a combination of zinc, a band 3 clustering agent, and BS^3^, a cluster-stabilizing agent (Zn/BS^3^). Band 3 clusters elicit a limited activation of the complement pathway and induce a predominantly complement-mediated RBC recognition and removal [12]. Both treatments induce maximal removal of RBCs [13].

#### Phagocytosis of anti-D IgG opsonized np-RBCs

**Figure 2:**
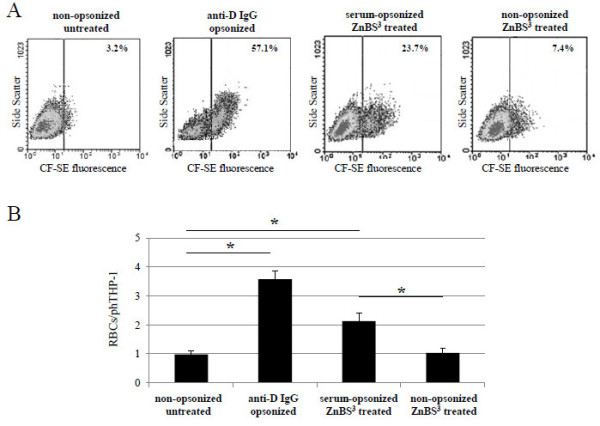
**Phagocytosis by THP-1 cells of positive control np-RBCs opsonized with anti-D IgG or treated with zinc/BS**^**3**^**. (A)** CF-SE fluorescence *vs* side scatter density plots showing THP-1 cells fed with non-opsonized untreated (left plot), anti-D IgG-opsonized (second plot), serum-opsonized, zinc/BS^3^-treated (third plot) and non-opsonized, zinc/BS^3^-treated (right plot) np-RBCs. Data are percentages of phagocytically active THP-1 cells above the fluorescence threshold set in each experiment. One representative experiment out of five with similar results. **(B)** Phagocytosis of variously treated positive control np-RBCs. Data are numbers of np-RBCs phagocytosed per phTHP-1 cell. Mean values ± SD (N = 5) ( *P < 0.001). For details, see Methods.

Phagocytosis of anti-D IgG opsonized np-RBCs was significantly (p<0.001) increased compared to non-opsonized np-RBCs, both as percentage of phTHP-1 cells from 3.2 ± 0.4% (non-opsonized, N = 5) to 56.9 ± 3.4% (anti-D IgG opsonized cells, N = 5), (Figure 2A, first and second dot plot, respectively), and as the mean number of cells phagocytosed per phTHP-1 from 1.0 ± 0.1(non-opsonized cells, N = 5) to 3.6 ± 0.3 (anti-D IgG opsonized cells, N = 5) (Figure 2B, first and second column, respectively).

#### Phagocytosis of Zn/BS^3^ treated np-RBCs

A second check with positive controls was performed with np-RBCs treated with Zn/BS^3^. Their phagocytosis was also significantly (P <0.002) increased compared to untreated np-RBCs, both as percentage of phTHP-1 cells from 3.2 ± 0.4% (non-opsonized untreated cells, N = 5 to 25.1 ± 3.2% (Zn/BS^3^ treated, serum-opsonized cells, N = 5) (Figure 2A, first and third dot plot, respectively), and as the mean number of cells phagocytosed per phTHP-1 from 1.0 ± 0.1 (non-opsonized untreated cells, N = 5) to 2.1 ± 0.3 (Zn/BS^3^ treated, serum opsonized cells, N = 5) (Figure 2B, first and third column, respectively, P <0.001). Control experiments demonstrated that serum opsonization is essential for phagocytosis induction, as Zn/BS^3^ treated, non-opsonized RBCs were not or poorly phagocytosed (Figure 2A, fourth plot, Figure 2B, fourth column).

#### Phagocytosis of stage-separated p- and np-RBCs single-labelled with CF-SE

**Figure 3:**
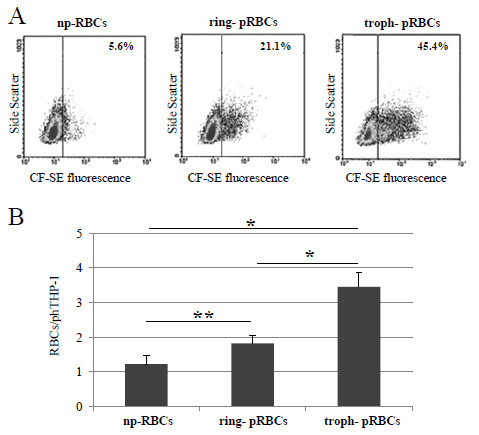
**Phagocytosis by THP-1 cells of stage-separated np- and p-RBCs single-labelled with CF-SE. (A)** Fluorescence *vs* side scatter density plots showing THP-1 cells fed with of serum-opsonized np-RBCs (left panel), ring-pRBCs (middle panel) and troph-pRBCs (right panel). Data are percentages of phagocytically active THP-1 cells above the fluorescence threshold set in each experiment. One representative experiment out of five with similar results. **(B)** Phagocytosis by THP-1 cells of CFDA-SE-labelled np-, ring- and troph-pRBCs. Data are numbers of RBCs ingested per THP-1 cell. Mean values ± SD (N = 5). Phagocytosis of troph-pRBCs *vs* np-RBCs and *vs* ring-pRBCs, and phagocytosis of ring- *vs* np-RBCs was significantly higher with P <0.001*, and P <0.02**, respectively. For details, see Methods.

Phagocytosis experiments were performed using synchronized ring- and troph-enriched fractions after Percoll gradient separation 20 hr and 40 hr after re-invasion, resulting in approximately 60% and >90% rings and trophozoites respectively. p- and np-RBCs from the same donor were stained with CF-SE, opsonized with human homologous serum (np-RBCs) and fed to THP-1 cells as detailed in Materials. Phagocytosis of p-RBCs increased stage-dependently either as percentage of phTHP-1 cells or as mean number of p-RBCs ingested per phTHP-1. phTHP-1 cells increased from 6.8 ± 1.7% (np-RBCs, N = 5) to 20.8 ± 2.2% (ring, N = 5) and 50.6 ± 4.4% (troph, N = 5) (Figure 3A, all P <0.001), while p-RBCs per THP-1 cell increased from 1.2 ± 0.3 (np-RBCs, N = 5) to 1.8 ± 0.2 (ring, N = 5), and to 3.5 ± 0.4 (troph, N = 5) (Figure 3B, troph *vs* ring and np-RBCs, P <0.001, ring *vs* np-RBCs, P <0.02).

#### Exclusion of artifacts

**Figure 4:**
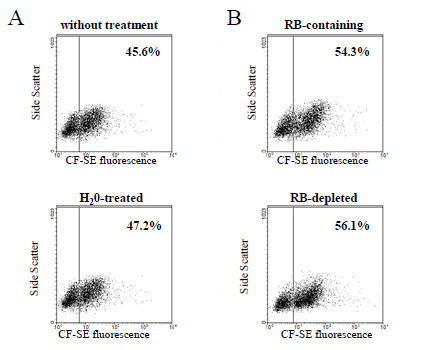
**Exclusion of artifacts caused by adherence of RBCs and residual bodies to THP-1 cells. (A)** Fluorescence of THP-1 cells fed with np-RBC-fed treated or not with water to lyse adherent intact RBCs. **(B)** Fluorescence of THP-1 cells fed with p-RBC in presence or absence of residual bodies (RBs). **(A, B)** CF-SE fluorescence *vs* side scatter dot plots of THP-1 cells fed with treated or untreated RBCs are very similar excluding artefactual adherence of RBCs or RBs to THP-1 cells. One representative experiment out of four **(A)** or three **(B)** with similar results. Data are percentages of phagocytically active THP-1 cells counted above the fluorescence threshold set in each experiment (for details, see Methods).

To exclude the presence of intact RBCs adherent to the THP-1 surface, ice-cold distilled water was added briefly to the cells after the phagocytosis period and immediately before FACS analysis. Physical parameters, fluorescence and phagocytosis data were not significantly changed by the treatment excluding the presence of intact RBCs adherent to the THP-1 surface (Figure 4A), indicating that the Ficoll passage after phagocytosis is sufficient to efficiently remove RBCs. Former observations with human monocytes fed with CF-SE labelled RBCs [14] indicate that no fluorescence was associated to membranes. Secondly, quenching activity by residual bodies (RBs), free haemozoin expelled from the mature schizont during merozoites release in the cultures was examined. RBs had no influence on phagocytosis quantification as indicated by invariant results shown in Figure 4B. Therefore, presented assay seems to be suitable for both RBs-depleted and RBs-containing cultures and for *ex vivo* analysis without further parasite separation.

#### Phagocytosis of p- and np-RBCs double-labelled with CF-SE and EB

**Figure 5:**
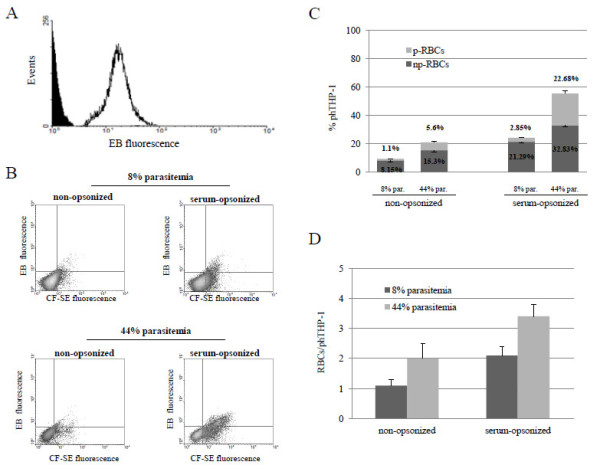
**Phagocytosis of troph-p-RBCs and np-RBCs double-labelled with EB and CF-SE. (A)** EB fluorescence of a synchronized trophozoite culture with 44% parasitaemia: troph-p-RBCs, black line, np-RBCs, full histogram. **(B)** CF-SE fluorescence *vs* EB fluorescence density plots of THP-1 cells fed with non-opsonized or serum-opsonized synchronized trophozoite cultures with 8% and 44% parasitaemia. In both upper (8% parasitaemia) and lower (44% parasitaemia) plots, upper and lower right quadrants show phagocytically active THP-1 cells fed with troph-p-RBCs or np-RBCs, respectively. Cells positive for CF-SE and EB were counted above the fluorescence threshold set in each experiment. One representative experiment out of six with similar results. **(C)** Phagocytosis of serum-opsonized and non-opsonized troph-p- and np-RBCs by THP-1 cells in synchronized trophozoite cultures with 8% and 44% parasitaemia. Numbers on bars are percentages of phagocytically active (ph)THP-1 cells. For each phTHP-1 value the share of p- and np-RBCs in the sample is indicated. Bar data are mean values of phTHP-1 cells ± SD (N=6). **(D)** Total phagocytosis of serum-opsonized and non-opsonized p- plus np-RBCs by THP-1 cells in synchronized trophozoite cultures with 8% and 44% parasitaemia. Data are numbers of p- plus np-RBCs phagocytosed per phTHP-1 cell. Bar data are mean values ± SD (N = 6). For details, see Methods.

In double-labelling experiments, np- and p-RBCs were labelled with ethidium bromide (EB), before CF-SE labelling. EB, a DNA intercalating agent, develops red fluorescence after binding to DNA. Therefore, EB was used to distinguish DNA-free np-RBCs from p-RBCs, which contain parasite DNA. Labelling conditions were optimized at 1 mg/ml EB, 10% haematocrit (1 × 10^6^ RBCs/μl) and 20 min incubation time. Figure 5A shows the EB fluorescence of a synchronized trophozoite cultures with 44% parasitaemia. The difference between np-RBCs and troph-pRBCs reflects absence and presence of DNA, respectively.

The aim of double staining with CF-SE and EB was to discriminate between phagocytosis of p- and np-RBCs in cultures containing both cell types, as only p-RBCs were stained by EB. FACS analysis of a THP-1 population fed with double-labelled RBCs clearly shows that p- and np-RBCs were phagocytosed in different proportions. Figure 5B-D shows the phagocytosis of two synchronized cultures at trophozoite stage and approximately 8% and 44% parasitaemia. Opsonization with homologous serum increased phTHP-1 from 9.25% and 20.9% (non-opsonized RBCs, 8% and 44% parasitaemia, respectively) to 24.14% and 55.51% (serum-opsonized RBCs, 8% and 44% parasitaemia, respectively). EB staining allowed to ascertain the share of p- and np-RBCs ingested at different parasitaemia values. As expected, at 44% parasitaemia the share of ingested p-RBC increased remarkably in both opsonized and non-opsonized samples. As shown in Figure 5C, 1.1% (8% parasitaemia) and 5.6% (44% parasitaemia) of phTHP-1 had phagocytosed at least one non-opsonized p-RBCs, while 2.85% (8% parasitaemia) and 22.68% (44% parasitaemia) of phTHP-1 had phagocytosed at least one serum-opsonized p-RBC. The mean number of RBCs (sum of p- plus np-RBCs) phagocytosed per phTHP-1 was also determined at low and high parasitaemia (Figure 5D).

To make sure that EB did not interfere with the phagocytosis quantification by CF-SE fluorescence, experiments were performed with np-RBCs stained with CF-SE only, or with both EB and CF-SE. Results indicated no significant difference between the two conditions (not shown). In cultures containing both p- and np-RBCs, quantification of phagocytosis using CF-SE labelling only did not discriminate between phagocytosed p- or np-RBCs. By contrast, additional DNA labelling by EB can discriminate between p- and np-cells and allowed to simultaneously quantify both the total number of RBCs ingested per phTHP-1 cell as well as the ratio between ingested p- and np-RBCs.

## Discussion

This study describes a rapid, sensitive, reproducible and accurate assay to quantify phagocytosis of p- and np-RBCs in the same sample using CF-SE- and EB-labelled target cells and the human phagocytic monocyte cell line THP-1. Phagocytes were previously activated with TNF and IFN*γ* to upregulate Fc- and complement receptors and enhance phagocytic efficiency without loss of specificity [10,11]. Target RBCs were double-labelled with CF-SE and EB. CF-SE is the fluorescent derivative of CFDA-SE, a non-fluorescent lipophilic molecule that passively diffuses into the cell where it is activated by esterase cleavage of its acetyl groups to the brightly fluorescent derivative CF-SE. The latter, a non-toxic molecule, is stably retained in the cell-forming covalent conjugates to free amino groups, emits stable and homogeneous fluorescence and does not interfere with RBC functionality [7,15]. CF-SE does not localize in cell membranes and does not elicit membrane alterations that may induce phagocytic recognition of the labelled cells. EB, a widely used DNA fluorescent label, allows to discriminate p-RBCs that contain parasitic DNA from the np-RBCs totally devoid of DNA.

The phagocytic efficiency of the THP-1 cells was checked with np-RBCs modified by two treatments known to maximally enhance phagocytosis: opsonization with anti-D IgG to stimulate IgG-mediated phagocytosis, and RBCs treatment with zinc-BS^3^, that generates stable band-3 clusters and induce a predominantly IgG/complement-mediated phagocytosis [9]. Phagocytosis by activated THP-1 cells was lower compared to phagocytosis by adherent human monocytes [9,16]. However, THP-1 cells displayed the same specificity and relative phagocytic activity with respect to p-RBCs as human monocytes, while presenting a number of advantages, such as independence from availability of human buffy-coats, constant performance, low cost and easy maintenance.

Removal of large numbers of np-RBCs accompanies the rupture of p-RBCs at schizogony, as shown by the simultaneous presence of p-and np-RBCs in the same phagocytic cell in peripheral blood and organ phagocytes [17-19] and by studies on the clearance kinetics of p- and np-RBCs [20,21]. It is generally maintained that np-RBCs are cleared in approximately 10-fold excess compared to elimination of p-RBC at schizogony. However, this estimate is based on a single study [5], performed by modelling 12 neurosyphilitic patients who underwent malaria therapy. Those authors observed a substantial destruction of np-RBCs occurring during two cycles of parasitaemia with peak values at 20,000-40,000 parasites per μl and estimated that an average of 8.5 np-RBCs were destroyed per rupturing schizont. An analogous study [22] has described an even larger excess removal of np-RBCs in *vivax* malaria anaemia, a frequent and severe complication of *vivax* infection occurring by largely undisclosed mechanism [23,24].

Apart from RBC lysis at schizogony, it is assumed that phagocytosis is the predominant mechanism of removal of np- and p-RBC. This assertion is based on a balance study in acute SMA where a quantitative comparison was performed between the loss of blood haemoglobin (Hb) and increase of Hb in plasma and urine. Increase in plasma Hb was less than 1% and excretion of Hb in urine was less than 0.5% of total Hb loss [25]. This data tends to exclude complement lysis [26] and is in line with the common paradigm of phagocytic removal of senescent, variously damaged RBCs and *falciparum* p-RBCs as the consequence of membrane damage and opsonization through enhanced binding of complement factor C3b and (anti-band 3)-IgG [9,27,28].

Most available cytofluorimetric methods (for example: [14,29]) do not allow to discriminate the relative contribution of np- vs p-RBC removal. One exception, however, is the method by Tippett *et al*[30] designed to assess phagocytosis by patient monocytes or by THP-1 cells of p- and np-RBCs simultaneously labelled by EB and fluorescein isothiocyanate (FITC). Comparison of our results with Tippett et al’ is difficult because they used unstimulated THP-1 cells with low phagocytic activity and FITC, a membrane-standing molecule known to interfere with RBC membrane transport systems and surface carbohydrates [31,32]. In few cases though, a comparison was possible. For example, the percentage of phagocytically active cells was between nil and 10% in unstimulated [30], and 45% in pre-stimulated [this study] THP-1 cells when both TPH-1 cells were challenged with serum-opsonized p-RBCs. Higher phagocytic activity was also noted when RBC suspensions with 3-8% p-RBCs were challenged by unstimulated [30] or pre-stimulated [this study] THP-1 cells. Phagocytically active cells were approx. 2% in unstimulated and approx. 24% pre-stimulated THP-1 cells.

In conclusion, due to the lack of reliable quantitative data on the relative role of phagocytosis of p- *vs* np-RBCs, the method presented here may help to fill gaps particularly in the pathogenesis of SMA. For example, it will be useful to analyse the factors or conditions that modulate the share of np-RBC removal *in vitro* and *in vivo*. It appears that np-RBC removal may vary within broad limits as documented in human SMA and in humanized murine models and thus be an important determinant of SMA [33,34]. The causes for this variability are unknown. Possible factors are bystander modifications that induce RBC membrane modifications, transfer of toxic parasite-produced molecules in rosettes [35], malaria- or age-related variations in phagocytosis-enhancing complement factors [26,36] and surface IgG [37], and upregulation of phagocytic activity of host phagocytes [20]. Phagocytosis studies *ex vivo* may also help to clarify the mechanism of malaria protection against severe anaemia observed in correlative studies in alpha-thalassaemia patients characterized by minimal alterations of RBC morphology, functionality and RBC lifespan in the np-status.

## Competing interests

The authors declare that they have no competing interests.

## Authors’ contributions

VG, PA, OAS and ES designed experiments; VG and OA performed experiments; VG and PA drafted the manuscript; all authors discussed experiments and results and commented on the manuscript. All authors read and approved the final manuscript.

## Supplementary Material

Additional file 1 **Optimization of phagocytosis assay. (A)** Dependence of phagocytosis on phagocytosis time. Cell ratio: 50 np-RBCs per THP-1 cell. **(B)** Dependence of phagocytosis on np-RBCs/THP-1 cell ratio. Phagocytosis time: 150 min. CF-SE-labelled, IgG anti-D-opsonized np-RBCs were exposed at indicated cell ratios to pre-activated THP-1 cells for various phagocytosis periods. Phagocytosis is expressed as percentage of phagocytically-active THP-1 (phTHP-1). Mean values ± SD (N = 4). For details, see Methods. Click here forn file
