# Supplementary figures and images for: Simultaneous determination of phagocytosis of Plasmodium falciparum-parasitized and non-parasitized red blood cells by flow cytometry
Source: Malar J. 2012 Dec 21;11:428. doi: 10.1186/1475-2875-11-428 (PMC3546840; doi:10.1186/1475-2875-11-428)

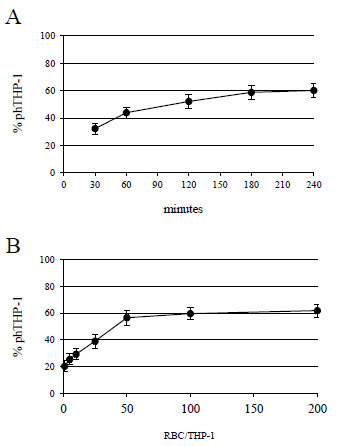

Supplement: Additional file 1 — Optimization of phagocytosis assay. (A) Dependence of phagocytosis on phagocytosis time. Cell ratio: 50 np-RBCs per THP-1 cell. (B) Dependence of phagocytosis on np-RBCs/THP-1 cell ratio. Phagocytosis time: 150 min. CF-SE-labelled, IgG anti-D-opsonized np-RBCs were exposed at indicated cell ratios to pre-activated THP-1 cells for various phagocytosis periods. Phagocytosis is expressed as percentage of phagocytically-active THP-1 (phTHP-1). Mean values ± SD (N = 4). For details, see Methods. [file 1475-2875-11-428-S1.jpeg]
